# Aseptic femoral nonunion treated with exchange locked nailing with intramedullary augmentation cancellous bone graft

**DOI:** 10.1186/s13018-022-03229-8

**Published:** 2022-07-06

**Authors:** Chi-Chuan Wu

**Affiliations:** Department of Orthopedic Surgery, Chang Gung Memorial Hospital, Chang Gung University, 5 Fu-Hsin St., 333, Guishan, Taoyuan, Taiwan

**Keywords:** Aseptic nonunion, Cancellous bone grafting, Exchange nailing, Femoral shaft

## Abstract

**Background:**

Closed reamed locked intramedullary nailing has been the treatment of choice for most of femoral shaft fractures. A high union rate with a low complication rate is generally predictable. For an aseptic femoral shaft nonunion with a prior inserted intramedullary nail, exchange nailing is one of favored surgical techniques for treatment. However, a greatly varied success rate of 72–100% has been reported. To improve the success rate of exchange femur nailing, a modified bone grafting technique was developed. The purpose of this retrospective study intended to evaluate outcomes of such a revised technique.

**Methods:**

From July 2011 to March 2019, 48 consecutive adult patients (average, 38 years; range, 19–67 years) with aseptic femoral shaft nonunions after intramedullary nailing treatment were studied. All femoral shaft fractures were initially caused by traffic accidents, which were treated by a closed or open intramedullary nailing technique at various hospitals. The current revision treatment was performed after an average of 2.2 years (range 1.1–6.2 years) from initial injuries. In the surgery, the prior nail was removed and the marrow cavity was reamed widely (at least 2 mm as possible). Sufficient cancellous bone grafts harvested on the trochanteric marrow wall from the inside were placed in the marrow cavity of the junction of nonunion fragments. A new 1-mm smaller size locked intramedullary nail was inserted. Whether the dynamic or static mode of nails were used mainly depended on the nonunion level. Postoperatively, protected weight bearing with crutches was allowed for all patients.

**Results:**

Forty-one patients were followed for an average of 2.8 years (85.4%; range, 1.9–4.5 years) and all fractures healed. The union rate was 100% (41/41, *p* < 0.001) with a union time of an average of 3.4 months (range, 2.5–5.0 months). There were no complications of deep infection, nonunions, malunions, implant failures or an avulsed trochanter tip fracture. The satisfactory knee function improved from 73.2% (30/41) preoperatively to 92.7% (38/41) at the latest follow-up (*p* = 0.019).

**Conclusions:**

The described modified bone grafting technique may effectively improve a union rate of exchange femur nailing while the surgical procedure is not complicated. It may therefore be used concomitantly in all aseptic femoral shaft nonunions when exchange nailing is performed.

## Background

A femoral shaft fracture is common and normally caused by high-energy injuries, especially, traffic accidents [1]. Currently, the treatment of choice for most of femoral shaft fractures is closed reamed locked intramedullary nailing and a high union rate with a low complication rate is generally predictable [2]. With such a technique, as high as the 94–98% union rate has been reported in the literature [3, 4].

A femoral shaft nonunion is uncommon, which may be caused by a number of etiologies [5, 6]. Clinically, a femoral shaft nonunion should be divided into the septic or aseptic type and the treatment principle is quite different [7]. A septic femoral shaft nonunion always requires staged surgeries during treatment. However, an aseptic femoral shaft nonunion can generally be treated with the one-time surgery. Although non-surgical treatment may be successful for an aseptic femoral shaft nonunion, in the literature surgical treatment with various techniques are still the mainstream [8].

Currently, two surgical techniques are widely used in the treatment of an aseptic femoral shaft nonunion [9]. The one is exchange nailing with a varied success rate of 72–100% [10]. The greatest advantage of this technique is its technical simplicity with a possibly high union rate. The second is plate augmentation with the prior nail in situ [11]. Although a higher union rate as compared to exchange nailing has been reported, placement of an augmented plate with or without bone graft supplementation always requires to create a big local wound. Imaginably, the surgical technique becomes more complicated and local complications including deep infection may potentially occur. Theoretically, if cancellous bone grafts can be supplemented much more, which greatly reinforce the osteogenic power, and local stability can be steadily maintained, the bone healing ability in exchanging nailing can be greatly upgraded [6, 12, 13]. Consequently, the union rate of exchange nailing may be greatly increased. Anatomically, the trochanteric marrow cavity is rich of cancellous bone which can be harvested along the nail tract from the inside. There is unnecessity to create another wound for transferring the cancellous bone graft. The purpose of this retrospective study intended to investigate the outcomes of such a modified cancellous bone grafting technique when exchange femur nailing is implemented.

## Methods

### Selection of patients

From July 2011 to March 2019, 48 consecutive adult patients (> 18 years) with aseptic femoral shaft nonunions, which had an intramedullary nail in the marrow cavity, were treated with the modified bone grafting technique at the author’s institution. The author singly performed the whole surgeries and followed all patients. Patients aged from 19 to 67 years (average, 38 years) with a male to female ratio of 3 to 1. All fractures were initially caused by motorcycle or car accidents and were treated at several different hospitals. There were no open femur fractures initially. The operation times were 1–4, which were performed at varied hospitals. However, nonunion persisted in all fractures (18 hypertrophic and 30 atrophic nonunions). All patients might or might not need crutches in the daily activities but a limp was evident if an aid was not used. The periods from the initial injuries to the current revision surgeries were on average 2.2 years (range, 1.1–6.2 years).

In the outpatients’ department (OPD), patient’s treatment histories were pursued in detail. Physical examination was performed to evaluate all associated injuries. Wound situations were evaluated by clinical manifestations, laboratory tests, and local plain radiographs. Associated leg length discrepancy (LLD) was detected by SMD (spino-malleolar distance) and FLSS (full-length standing scanogram) measures. For patients with suspicion of deep infection, staged operations were advised. For patients with LLD of more than 1.5 cm, concomitant femur lengthening was performed [14]. Patients with the two situations were excluded from the current study. Inclusion criteria for this study were adult patients (> 18 years), a femoral shaft nonunion alone, and a prior intramedullary nail in situ. Exclusion criteria were wound infection history, more than 1.5 cm of LLD or associated knee injuries.

### Surgical technique

Under generalized intubated anesthesia, the patient was placed on the operating table in the lateral decubitus position. An image intensifier was prepared for use.

**Fig. 1:**
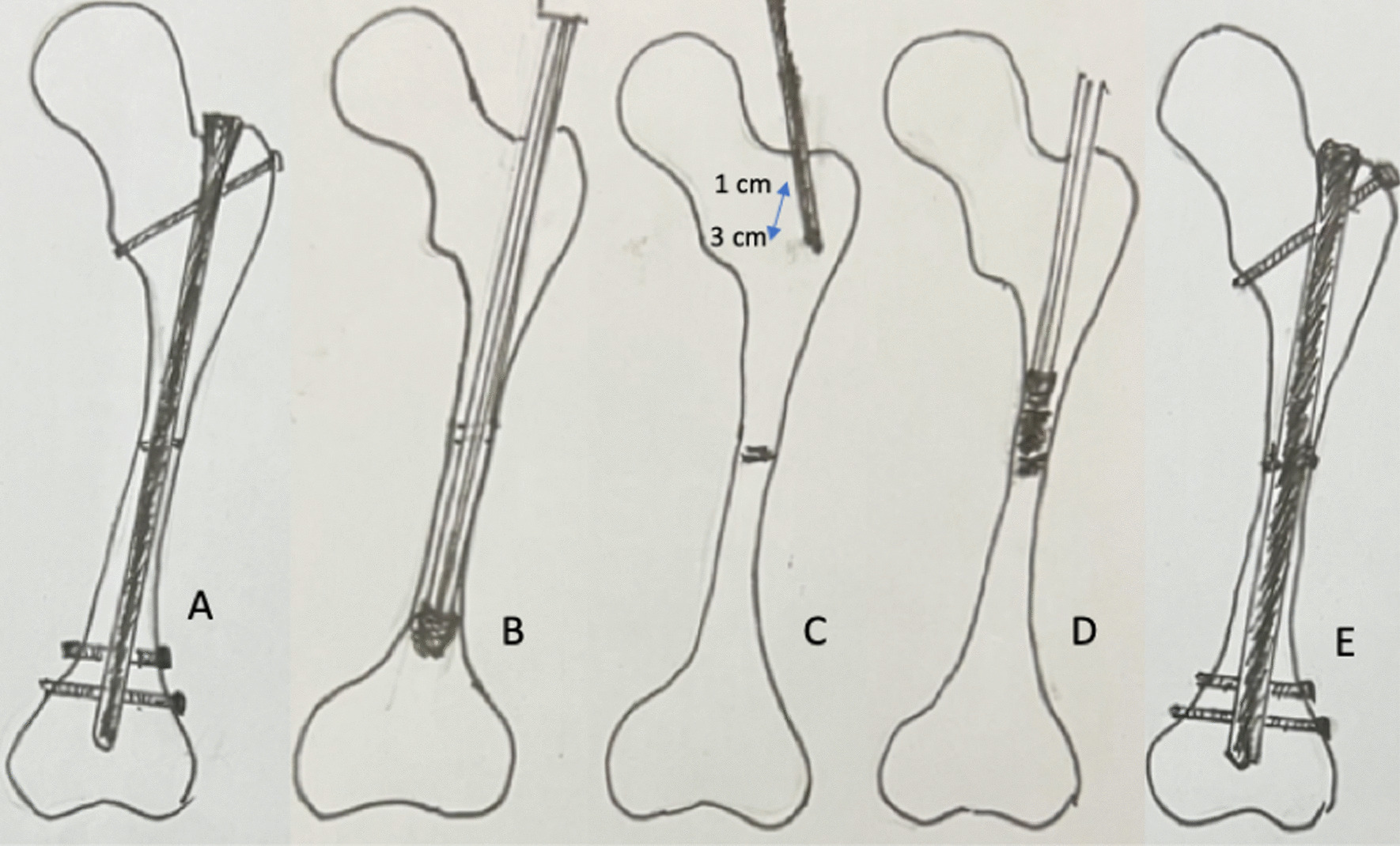
Sequence of exchange nailing with modified bone grafting is demonstrated. **A** An aseptic femoral shaft nonunion is treated. **B** Wider reaming is performed after removal of the prior nail. **C** Trochanteric cancellous bone graft is harvested through the nail inlet with a straight gouge. **D** Harvested cancellous bone graft is pushed at the proximal bone segment around the nonunion site. **E** A new locked nail is inserted

The prior intramedullary nail (including the broken nail) was removed completely (Fig. 1A). The residual implants including cerclage wires or broken screws were left in situ. For a locked nail with incarcerated broken screws, the screw shank was pushed forward with a smaller screw driver. For mal-aligned fragments, forceful manipulation was implemented to restore the acceptable axis (Open reduction to restore the acceptable fragment alignment was always unnecessary. With the previous intramedullary nail in situ, the previous malalignment was generally not prominent. As long as the previous intramedullary nail was removed, the nonunion site would be oscillating). Then, a rigid guide wire was inserted to create a new well-aligned canal (aiming at the trochlear groove). The size of the prior intramedullary nail was checked. The marrow cavity was reamed to at least 2 mm wider than the prior intramedullary nail size as possible (Fig. 1B).

To harvest sufficient amount of cancellous bone grafts, a smaller size straight gouge was inserted from the nail inlet and reached the marrow cavity of the trochanter (Fig. 1C). The wall surrounding the canal was longitudinally dug with 1 to 3 cm long from the nail inlet. The amount of cancellous bone grafts harvested was based on the nonunion volume (usually, 2–5 ml). Consequently, the cancellous bone grafts were pushed to the proximal bone segment around the nonunion site with a smaller size reamer under the guidance of an image intensifier (Fig. 1D).

A 1-mm smaller size locked nail (Russell-Taylor locked nail, Smith & Nephew Inc., Memphis, TN, USA) was inserted for definite stabilization (Fig. 1E). The mode of dynamic or static assembly completely depended on the nonunion level. A dynamic locked nail was normally preferred. However, a locked nail with the insufficient size (2 mm smaller than the reaming) had to be used with the static assembly. For the femur with shortening of 1–1.5 cm, a static locked nail was also required to prevent further shortening [14].

### Postoperative treatment

Postoperatively, patients were allowed to ambulate as early as possible. Walking with partial weight bearing using crutches was advised until the fracture healed. Knee and ankle range-of-motion (ROM) exercises were encouraged in the daily activity. Patients were followed-up at the OPD in the intervals of 4–6 weeks. Clinical and radiologic fracture healing processes were recorded. Complications were treated whenever necessary. After the fracture healed, patients were followed annually and the nail was advised to be removed at least 2 years later.

A fracture union was defined as clinically, no pain, no tenderness, and walking without aids; Radiographically, solid callus having connected three out of four cortices at anteroposterior and lateral radiographs [15, 16]. A nonunion was defined as the fracture site was still unhealed after 9-month treatment or a second surgical procedure was necessary to achieve a union [6, 17].

The knee function was evaluated by combinations of pain, stability, and ROM. Four grades were divided and a satisfactory outcome included an excellent or good grade. An excellent grade included no pain, intact stability, less than 5 degrees of flexion contracture, and more than 120 degrees of knee flexion. A good grade included no pain, minimal instability, less than 5 degrees of flexion contracture, and more than 90 degrees of knee flexion. A fair grade included mild pain, mild instability, less than 10 degrees of flexion contracture, and 70–90 degrees of knee flexion. A poor grade did not evaluate pain and instability but focused on more than 10 degrees of flexion contracture or less than 70 degrees of knee flexion.

### Statistical analysis

For the convenience of comparison, the Chi-square test was used for non-continuous data. The statistical significance was set at *p* < 0.05.

## Results

**Fig. 2:**
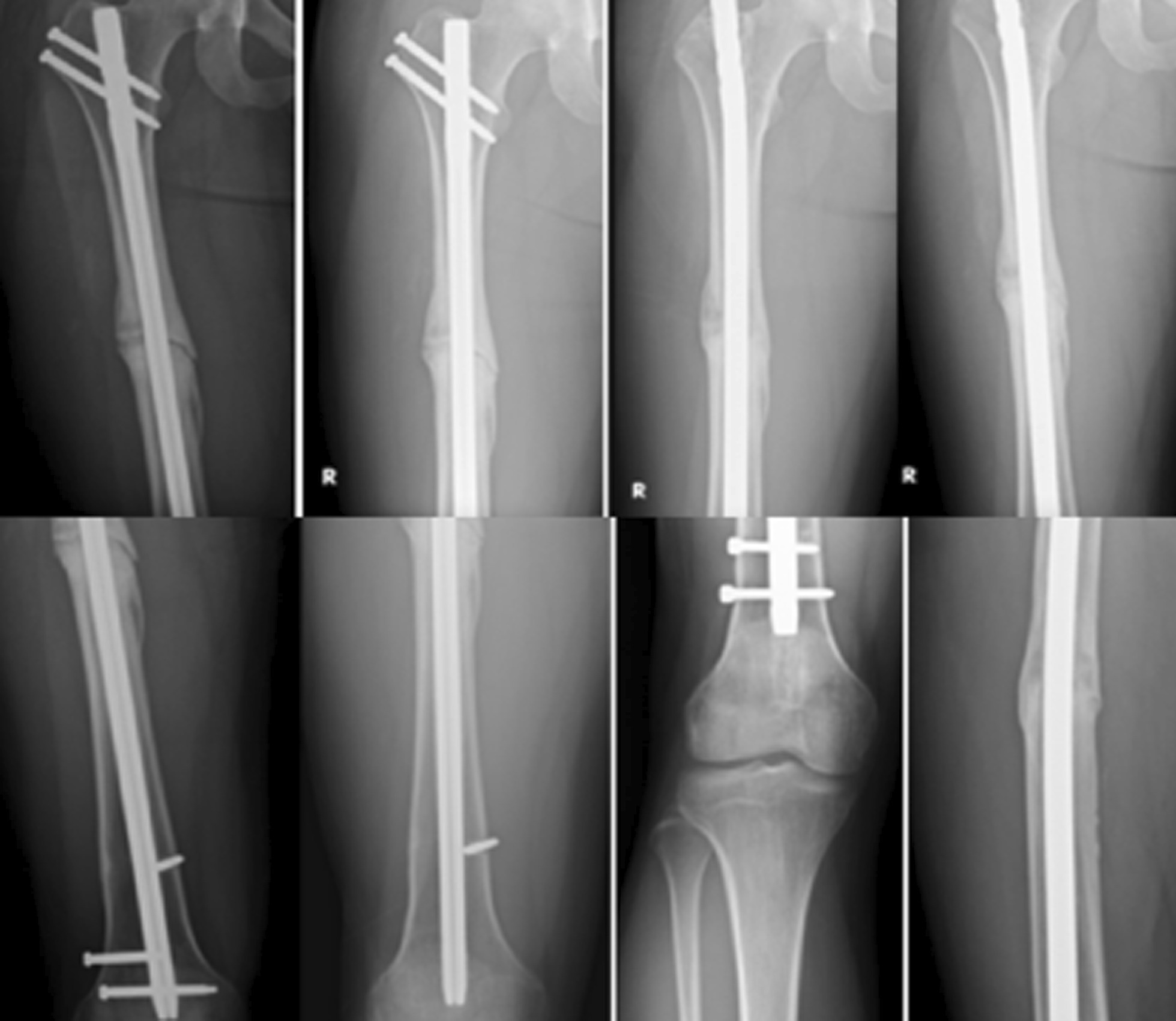
A 32-year-old man sustained a right aseptic femoral shaft nonunion for 2 years. Although distal dynamization for the static locked nail was performed, the nonunion persisted for 2 years. Exchange nailing of dynamic assembly with modified bone grafting caused solid union at 4 months

**Fig. 3:**
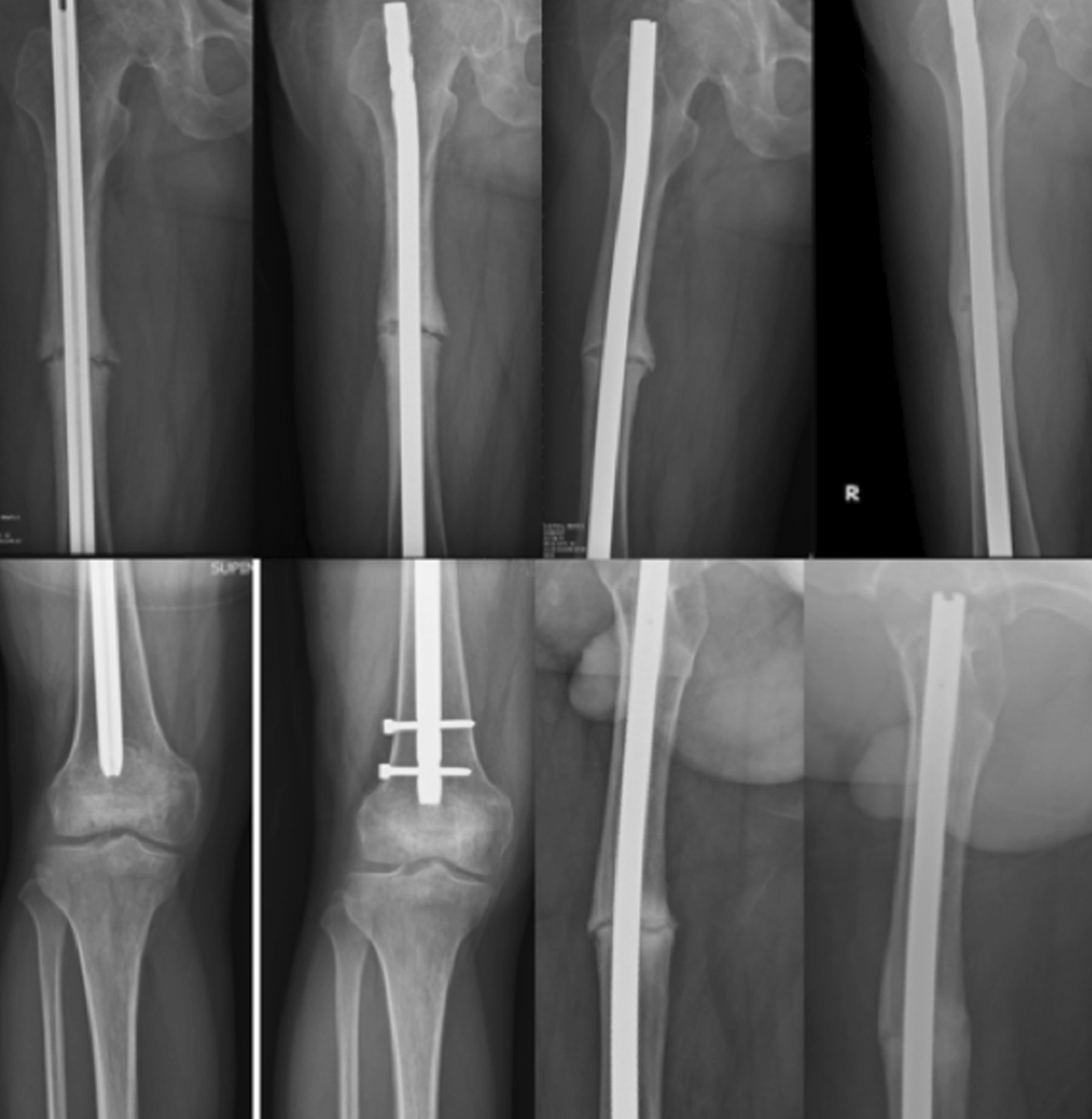
A 42-year-old man sustained a right aseptic femoral shaft nonunion for 2 years. The prior Kuntscher nail was removed. Exchange nailing of dynamic assembly with modified bone grafting caused solid union at 3.5 months

**Fig. 4:**
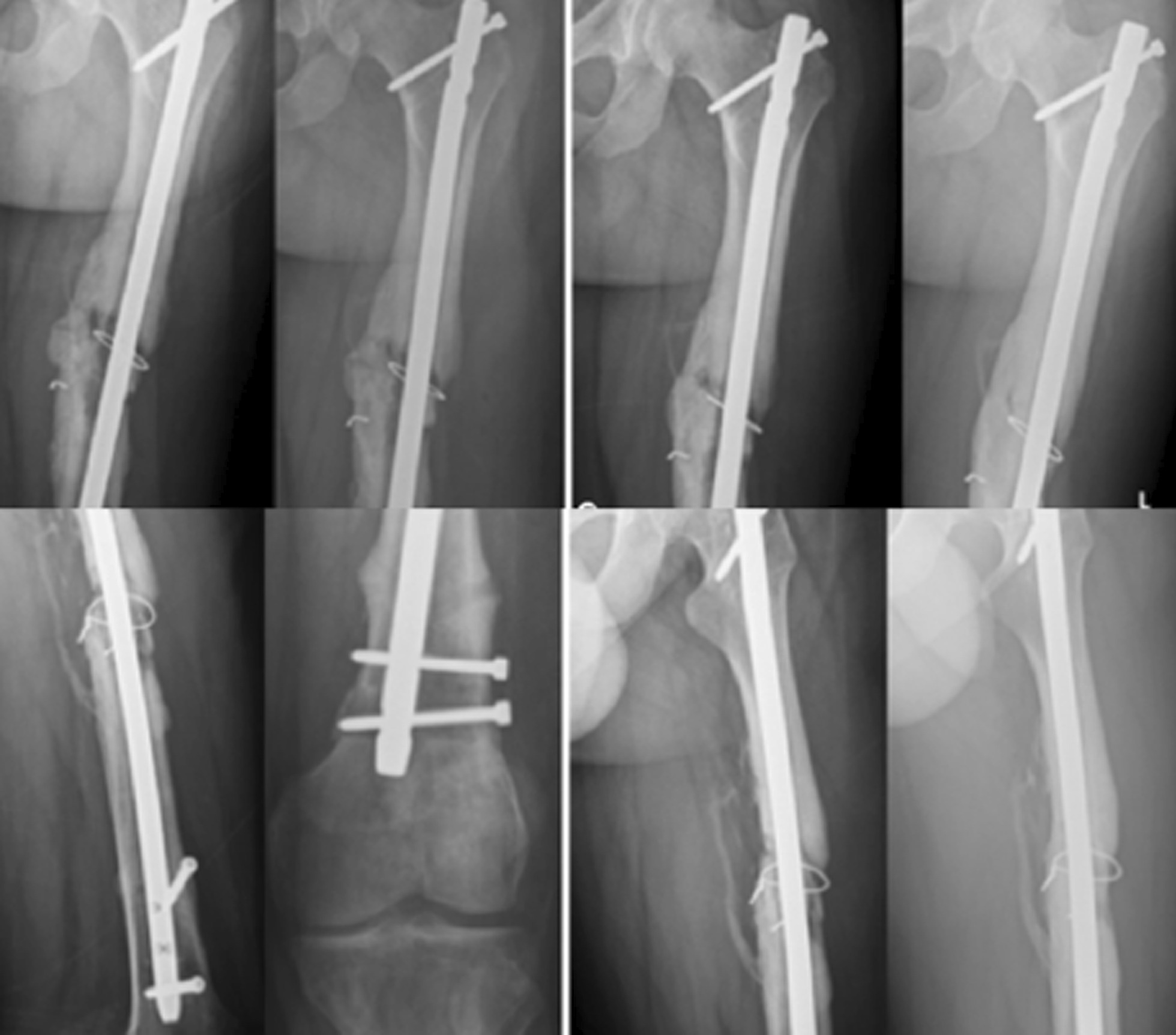
A 52-year-old man sustained a left aseptic femoral shaft nonunion for 3 years. Exchange nailing of static assembly with modified bone grafting caused solid union at 4.5 months

**Fig. 5:**
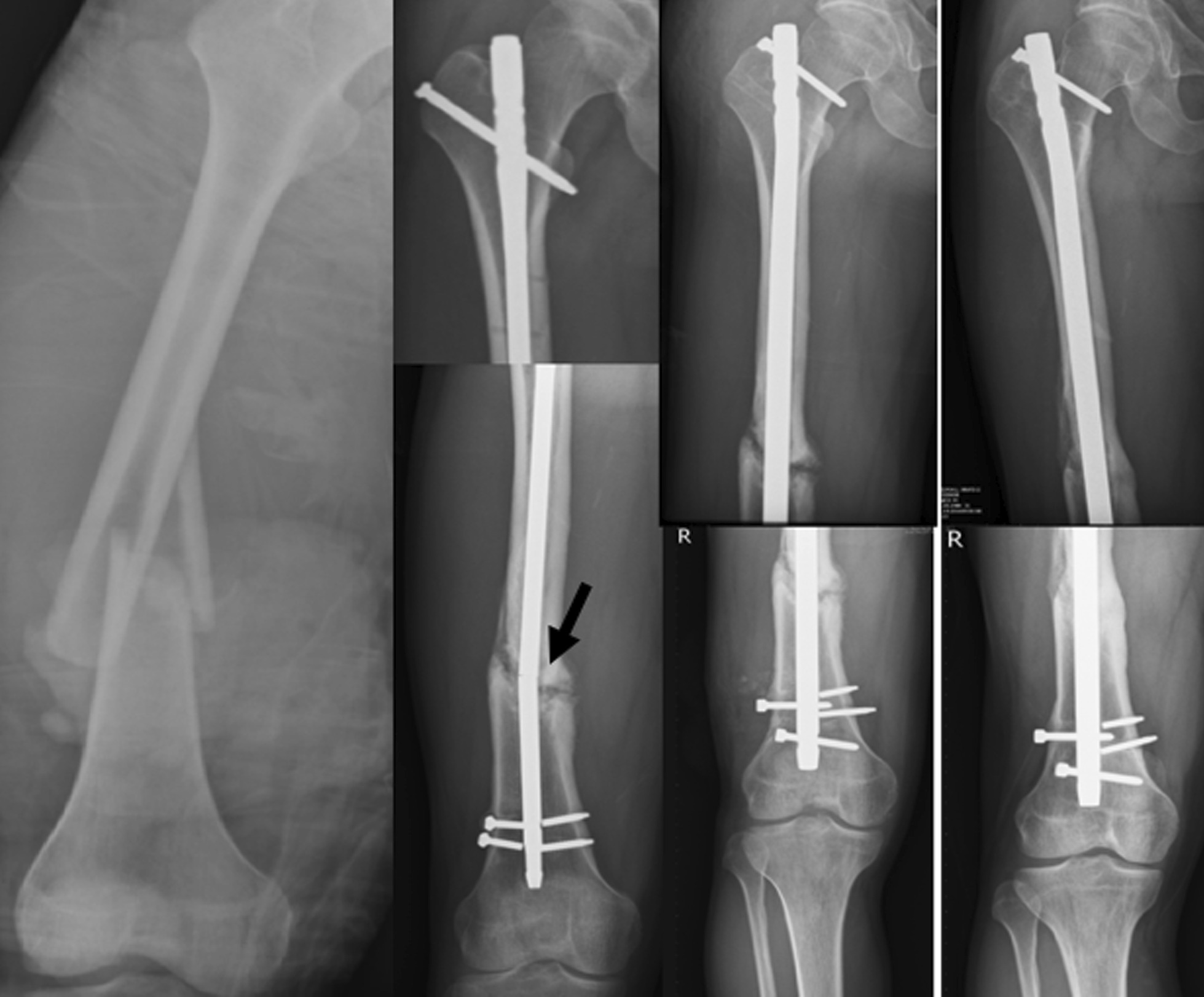
A 34-year-old man sustained a right distal femoral shaft comminuted fracture and was treated with closed static locked nailing. An aseptic nonunion with breakage of the nail (arrow) and both distal locked screws occurred at 2.5 years. Exchange nailing of static assembly with modified bone grafting caused solid union at 4 months

Forty-one out of 48 patients were followed for at least 9 months (85.4%; average, 2.8 years; range, 1.9–4.5 years; Table 1). Seven patients were lost despite all efforts to contact them. All 41 nonunions healed with a union rate of 100% (41/41, *p* < 0.001) and the union time of on average 3.4 months (range, 2.5–5.0 months; Figs. 2, 3, 4, 5).

**Table 1:** Demography and outcomes of patients with an aseptic femoral shaft nonunion treated by exchange nailing and modified cancellous bone grafting (*n* = 41)

Items	Description
Age (year)	Average, 38; range, 19–67
Gender (male/female)	31/10
Time from initial injury (year)	Average, 2.2; range, 1.1–6.2
Union rate (%)	100 (41/41)
Union time (months)	Average, 3.4; range, 2.5–5.0
Complications	0
**Knee function grade**	
Excellent	78.1% (32/41)
Good	14.6% (6/41)
Fair	4.9% (2/41)
Poor	2.4% (1/41)
**Knee flexion**	
> 120°	78.1% (32/41)
> 90°	14.6% (6/41)
> 70°	4.9% (2/41)
< 70°	2.4% (1/41)
Follow-up (year)	Average, 2.8; range, 1.9–4.5

________________________________________________________________________

Wider reaming of 2 mm with 1-mm less size locked nails were used in 28 patients (68.3%). Wider reaming of 1 mm with the same size locked nails were used in seven patients (17.1%). Six patients (14.6%) had previous nails more than the largest size of 1 mm achievable at the author’s institution. Thus, a 2-mm smaller size locked nail was used. A static locked nail had to be used in the second and the third groups to maintain the stability.

There were no nonunions, malunions, deep infection, implant failures or an avulsed trochanter tip fracture.

A satisfactory knee function improved from 73.2% (30/41) preoperatively to 92.7% (38/41) at the latest follow-up (*p* = 0.019). The 38 patients with satisfactory knee function included 32 excellent results (78.1%, 32/41) and 6 good results (14.6%, 6/41). Three patients with unsatisfactory knee function were due to less than 90 degrees of knee flexion (Table 1). Judet quadricepsplasty to improve the knee flexion was therefore advised to the three patients whenever they considered the requirement [18].

The intramedullary nails were removed for 35 patients (85.4%, 35/41). Generally, patients were advised to remove the nail for avoidance of stress concentration and shielding, which might weaken the femur strength in the long-term period [19].

## Discussion

Factors favoring fracture healing are minimal gap, adequate stability, and sufficient nutrition supply [20]. In 2007, the diamond concept for fracture healing was advocated [21]. The four factors in the diamond concept including scaffold, mechanical stability, mesenchymal stem cells, and growth factors are similar to the traditional concepts. Integrating both theories had emphasized the two decisive mechanisms: biomechanical and biological factors in the treatment of a fracture [22, 23]. Once either of the two mechanisms is defective, a nonunion may occur. Similarly, treatment of a nonunion must create the optimal condition for both mechanisms to upgrade the success rate [5, 13].

Because open femoral shaft fractures are relatively few (due to surrounded massive muscle masses), with closed locked intramedullary nailing treatment, femoral shaft infection is uncommon. Generally, femoral shaft nonunions are aseptic. Contrarily, tibial shaft fractures are often open due to its anteromedial aspect is only skin-covered. Therefore, a septic nonunion is very common [24, 25]. In the literature, the treatment principle had been largely established. Because muscles around the tibia is not massive, either external fixation or internal fixation for septic tibia can be successful. On the other hands, the femur is deeply seated. Continuous drain of infected hematoma after revision surgery is often less effective. Recurrence is common with one-stage treatment in septic femur nonunions [7].

Practically, an aseptic nonunion should be divided into the hypertrophic or atrophic type because each is caused by the different patho-mechanisms [26]. A hypertrophic nonunion is usually caused by insufficient biomechanical stability and massive callus can be found on the plain radiographs. The treatment of choice is providing sufficient local stability. An atrophic nonunion is generally caused by loss of osteogenic power, which is due to segmental bone defects, comminuted fractures or destructive local vascularity. A rational treatment must greatly initiate osteogenic potential. Clinically, exchange femur nailing is believed to be able to provide favorable factors to reinforce both mechanisms [27]. However, radiographic identification is sometimes difficult. Exchange nailing may achieve insufficient amount of cancellous bone graft. It may be one of failed reason in exchange nailing. In the present study, all nonunions are added with cancellous bone graft to promote osteogenic potentials and healed totally.

In the literature, reaming the marrow cavity with a larger size reamer is considered to remove local scar tissues surrounding the nonunion site. With destroying the intramedullary blood vessel connection by reaming, the growth factors are released from the disrupted blood vessels [9, 13, 28]. Theoretically, if the local stability can be affirmed, the fracture healing process should be predictable. In the literature, a greatly varied success rates are reported, 72–100% [10]. Technical variations, small sample sizes, and different patients’ conditions (different severity of prior surgical damages) may produce the quite different outcomes [29].

Because an aseptic femoral shaft nonunion is uncommon and factors hinder the fracture healing process are multiple, accurate determination of unfavorable factors may be difficult [5]. Under such situations, surgical techniques which may concomitantly cover both favorable mechanisms while they will not greatly produce technical complexity are valuable practically. In the current study, a modified bone grafting technique was developed. The outcomes are considered quite successful.

Anatomically, the femur in different persons possesses varied degrees of anterior bowing, which may be unfit for anterior bowing of the locked femur nail [30]. Thus, the nonunion site may be not nail-cortical contact tightly between the nail and the canal. The current study supplements the cancellous bone graft from the inside, which may partially reinforce the local stability for fracture healing [31]. The harvested cancellous bone graft is placed and pushed by a smaller size reamer at the proximal bone segment around the nonunion site. The inserted locked nail which is 1-mm smaller than the finished reaming will have a space to keep the cancellous bone graft. If the nonunion site has a large bone defect, it can keep the cancellous bone graft more efficiently (Fig. 4).

Harvesting cancellous bone graft from the greater trochanter for nearby bone grafting has been used for several decades (e.g., cancellous bone graft for subtrochanteric nonunion or malunion). As long as the harvested sites are not too close to the greater trochanter tip (in the present series, 1–3 cm to the cortex is advised) with protected weight bearing after revision surgery, the greater trochanter stability should be sufficient (Fig. 1). After 3 months of post-surgery, cancellous bone healing should be completed. No greater trochanteric avulsion fractures were noted in the present series.

In the current study, a dynamic locked nail is preferred first and a static locked nail is indicated for reinforcing local stability or prevent progressive shortening [13]. Biomechanically, a dynamic locked nail is the load-sharing device, which lets the loads partially transfer along the femoral cortex [32]. Therefore, implant failure can be reduced and fracture healing process can be promoted. On the contrary, a static locked nail is the load-bearing device [33]. The loads completely transfer via the screws and nails. Implant failure may occur if protected weight bearing is neglected. In the current study, all fractures healed and there was no implant failure.

Theoretically, the traditional exchange femur nailing still has a few traps, which may lower a success rate. A number of femoral shaft fractures have previously been stabilized with the largest intramedullary nails but still become nonunions. Removal of the previous nail and reaming the femoral canal 1 mm wider will have no suitable new nails of 1-mm smaller size. Under such situations, cancellous bone grafts procured from the trochanteric wall from the inside may provide sufficient amounts of bone grafts to promote osteogenesis. The 2-mm smaller size locked nail as compared to the wider reaming must use the static mode of nail for reinforce the local stability [34]. In the current study, all 6 patients (14.6%) could achieve a solid union within 5 months.

In the current study, the knee function grades were re-defined (Table 1). Except that the pain and the stability are maintained as original levels, knee ROM is relaxed [35]. In the modern life, knee flexion with more than 90 degrees, driving a car or riding a motorcycle is not inconvenient. For knee flexion more than 120 degrees, all daily activities will be undisturbed. Thus, it is unnecessary for patients with complete squatting (140 degrees of knee flexion). Performing quadricepsplasty to gain a few additional knee flexions maybe less cost-effective.

In the literature, a success rate with exchange nailing for treatment of aseptic femoral shaft nonunions has been advancing years by years. It should be due to well-developed modern medical concepts and advanced technology. Both mechanisms for promoting fracture or nonunion treatment have achieved epochal amelioration and innovation [27]. Because exchange nailing only requires a small incision wound following the prior surgery, it should become the treatment of choice after the success rate is further upgraded. The present report describes a simple and effective cancellous bone graft harvesting technique. It does not need to create other wounds. Therefore, the surgical time and complications may be improved. Theoretically, the osteogenic potential from the described technique will not be superior to cancellous bone graft harvested from other sites.

The limitations of this study may occur. The first, there were no comparative groups for comparing the superiority of traditional exchange nail and the current modified technique. After all, an aseptic femoral shaft nonunion is uncommon. For more than 8 years, only 48 patients were treated. Dividing the 48 patients into two groups for comparison, statistical significance is difficult to be completed. The current study used a retrospective case series to complete the whole course. Based on theoretical and clinical considerations, the current study should be believable. The second, whether the described technique is better than plate augmentation is also not compared. The advantages of exchange nailing are its technical simplicity and associated surgical complications are normally negligible. The current study tried the best to develop a technique to upgrade the success rate of exchange femur nailing. The feasibility may be greatly helpful for indicated patients.

## Conclusion

The described modified bone grafting technique may effectively improve a union rate of exchange femur nailing while the surgical procedure is not complicated. It may therefore be used concomitantly in all aseptic femoral shaft nonunions when exchange nailing is performed.

## Data Availability

Not applicable.

## Abbreviations

FLSS: Full-length standing scanogram
LLD: Leg length discrepancy
OPD: Out-patients’ department
ROM: Range of motion
SMD: Spino-malleolar distance
